# Distribution of mesopredatory fish determined by habitat variables in a predator-depleted coastal system

**DOI:** 10.1007/s00227-016-2977-9

**Published:** 2016-09-07

**Authors:** Lena Bergström, Martin Karlsson, Ulf Bergström, Leif Pihl, Patrik Kraufvelin

**Affiliations:** 1 Department of Aquatic Resources, Institute of Coastal Research, Swedish University of Agricultural Sciences, Skolgatan 6, 74242 Öregrund, Sweden; 2 Department of Biological and Environmental Sciences, Gothenburg University, Kristineberg 566, 45178 Fiskebäckskil, Sweden; 3 Environmental and Marine Biology, Department of Biosciences, Åbo Akademi University, Artillerigatan 6, 20520 Turku, Finland

## Abstract

Shallow nearshore habitats are highly valued for supporting marine ecosystems, but are subject to intense human-induced pressures. Mesopredatory fish are key components in coastal food webs, and alterations in their abundance may have evident effects also on other parts of the ecosystem. The aim of this study was to clarify the relationship between the abundance of coastal mesopredatory fish, defined as mid-trophic level demersal and benthic species with a diet consisting predominantly of invertebrates, and ambient environmental variables in a fjord system influenced by both eutrophication and overfishing. A field survey was conducted over a coastal gradient comprising 300 data points sampled consistently for fish community and environmental data. Results from multivariate and univariate analyses supported each other, demonstrating that mesopredatory fish abundance at species and functional group level was positively related to the cover of structurally complex vegetation and negatively related to eutrophication, as measured by water transparency. Contrary to other studies showing an inverse relationship to piscivore abundance over time, the spatial distribution of mesopredatory fish was not locally regulated by the abundance of piscivorous fish, probably attributed to piscivores being at historically low levels due to previous overfishing. Mesopredatory fish abundance was highest in areas with high habitat quality and positively related to the abundance of piscivores, suggesting a predominance of bottom-up processes. We conclude that, in parallel with ongoing regulations of fishing pressure, measures to restore habitat function and food web productivity are important for the recovery of coastal fish communities in the area.

**Electronic supplementary material:**

The online version of this article (doi:10.1007/s00227-016-2977-9) contains supplementary material, which is available to authorized users.

## Introduction

Shallow nearshore habitats are highly valued components of the marine ecosystem, upholding a wide range of regulatory and provisioning ecosystem services (Rönnbäck et al. 2007; de Groot et al. 2012; Costanza et al. 2014). Fish in shallow coastal habitats contribute to food web functioning and also directly support commercial and recreational fisheries (Seitz et al. 2014). In parallel, the coastal habitats are subject to strong pressure from human activities, in addition to being shaped by natural environmental gradients (Airoldi and Beck 2007; Halpern et al. 2008).

**Fig. 1:**
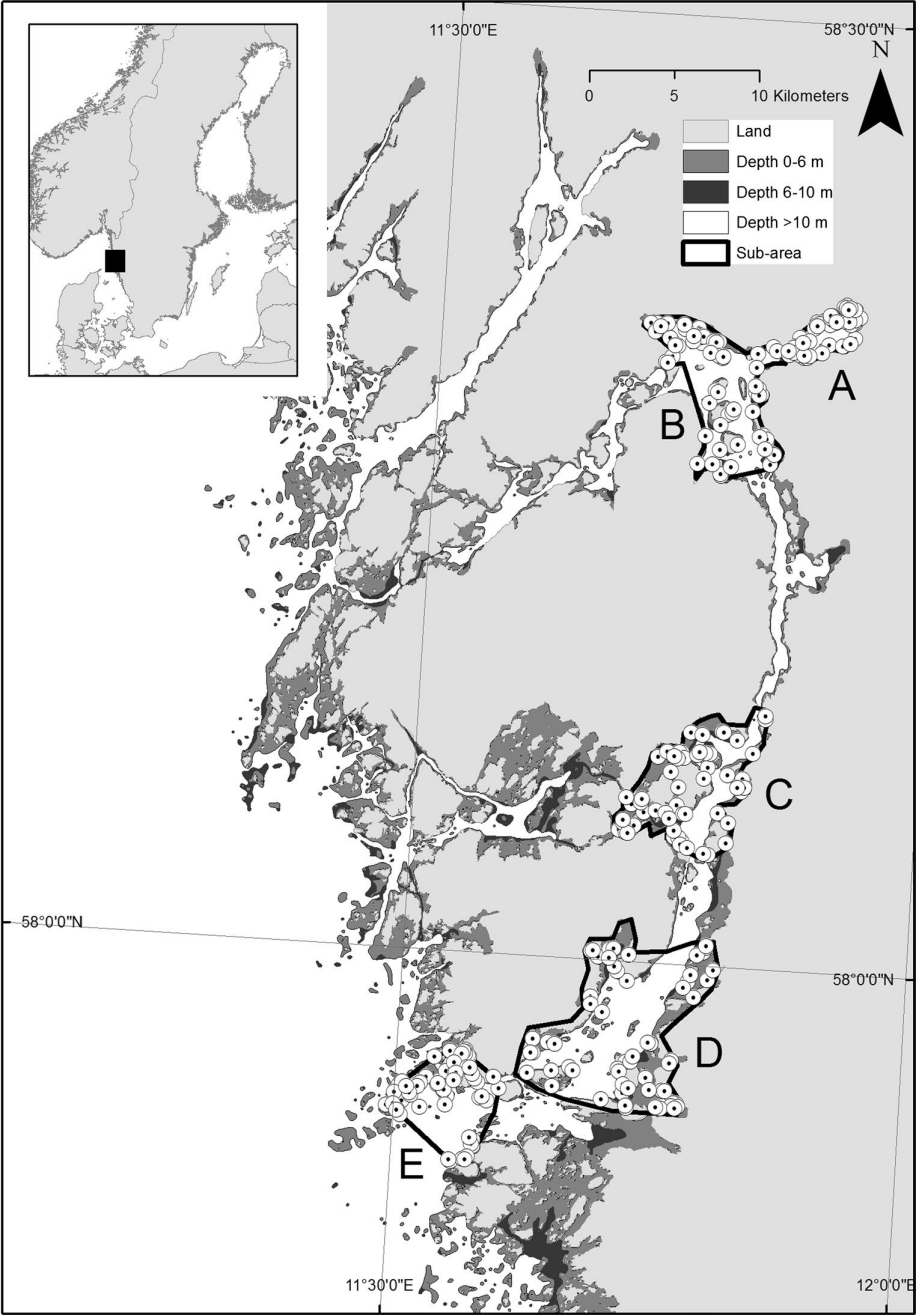
Map over the study area on the Swedish west coast. Sampling stations are denoted with *symbols*. *A*–*E* denote subareas referred to in the text. Sixty stations were sampled within each subarea

The most prominent examples of human-induced impact in temperate coastal areas include over-fishing and nutrient enrichment. In the Skagerrak, NE North Sea (Fig. 1), fishing pressure has historically been high on offshore as well as coastal populations. Many piscivorous fish populations are depleted or locally extinct, although restrictive management measures have been implemented more recently (Svedäng and Bardon 2003; Cardinale and Svedäng 2004; Casini et al. 2005; Stål et al. 2008). The abundance of mesopredatory fish, such as gobies and labrids, has been observed to increase the past three decades, concurrently with a decrease in large piscivorous fish attributed to the effects of fisheries (Pihl and Wennhage 2002; Svedäng and Bardon 2003; Gjøsæter and Paulsen 2004; Eriksson et al. 2011). Similar changes in the relative dominance of different functional groups are well known also in other marine systems and are explained by predation release on lower trophic levels due to a fisheries-induced depletion of piscivorous fish (Pauly et al. 1998; Jackson et al. 2001; Daan et al. 2005; Britten et al. 2014). Examples of such cascading effects are particularly common in offshore areas (Frank et al. 2005; Casini et al. 2008; Möllmann et al. 2009) but increasing also for coastal areas (Eriksson et al. 2009; Estes et al. 2011; Byström et al. 2015). At the same time, the Skagerrak area has been affected by eutrophication, which has been expressed in coastal areas as a shift from habitats dominated by eelgrass *Zostera marina* and sugar kelp *Saccharina latissima* to domination of ephemeral algae, with effects on the spawning and breeding conditions of coastal fish and hence potentially on the productivity base of the coastal food web (Baden et al. 2003; Moy and Christie 2012).

Quite importantly in this context, the ecological expressions of fisheries and eutrophication may also enhance each other. For example, cascading top-down effects may lead to reduced grazing control and cause further impaired eutrophication symptoms in coastal areas, such as increased biomasses of ephemeral algae (Eriksson et al. 2011). Moksnes et al. (2008), for instance, showed possible connections between overfishing of cod and increased populations of smaller fish species (gobies), which consumed mesograzers (mainly amphipods) down to levels causing excessive production of ephemeral algae, ultimately leading to further impaired habitat quality. In a meta-analysis covering studies in the North Atlantic, Östman et al. (2016) estimated that small fish species, through their regulatory effects on grazing amphipods and isopods, on average doubled the biomass of ephemeral algae compared to conditions where these fish were excluded. Fish in the lower and middle parts of the food web, commonly referred to as mesopredatory fish, thus have a central role in these processes. The local abundance of mesopredatory fish may potentially reflect environmental changes via both bottom-up (i.e. changes in habitat quality) and top-down (i.e. changes in predation pressure) mechanisms. Consequently, the abundance of mesopredatory fish may also have evident effects on other parts of the food web, by serving as a food source for higher trophic levels or by having a regulating function on lower trophic levels (Sieben et al. 2011; Baden et al. 2012; Östman et al. 2016).

In the current study, we aim at exploring the relationship between local mesopredator abundance and ambient natural and human-induced environmental pressures. We define coastal mesopredatory fish as mid-trophic level demersal and benthic species with a diet consisting predominantly of invertebrates. One important aim of the study is, however, to explore the applicability of this definition in a management context, to see whether this broadly defined functional group displays a homogenous response to ambient environmental factors or not. The study is highly motivated for increasing our general understanding of the relationships of key coastal species and functional groups to human-induced pressures and in order to identify relevant surveillance indicators to support an ecosystem-based management. The relevance of the study is additionally enhanced by the fact that some mesopredatory fish species have recently gained increased interest for fishery. The direct fishing pressure on coastal mesopredatory fish is generally low in Swedish coastal areas, but labrids (such as goldsinny wrasse, *Ctenolabrus rupestris*; corkwing wrasse, *Symphodus melops*; ballan wrasse, *Labrus bergylta*) are increasingly exploited for use in fish farms to delouse farmed salmon from sea lice (Darwall et al. 1992; Skiftesvik et al. 2014). Hence, a documentation of species–environment relationships in this area, prior to potential influence from this newly emerged fishery, is highly warranted.

Our objectives were to assess the relative importance of different environmental variables for the abundance of mesopredatory fish. We address the objectives by analysing a vast fish survey data set from a coastal fjord-like ecosystem in the Skagerrak, eastern North Sea, which encompasses spatial differences in both eutrophication and fishing pressure, as well as natural environmental gradients. With reference to previous studies (see above), we hypothesise that mesopredatory fish abundance will:

- Increase in areas with decreased piscivore abundance, signalling mesopredator release and/or
- Decrease in areas with higher eutrophication and reduced cover of structurally complex vegetation, signalling effects of habitat deterioration on fish productivity.

The hypotheses were evaluated in relation to the alternative hypothesis that mesopredator abundance is not affected by these biotic interactions, but is merely explained by variation in natural abiotic environmental factors, such as temperature, salinity, wave exposure, and depth.

## Materials and methods

### Study area

The studied area is a fjord-like system with several interconnected water bodies in Skagerrak on the Swedish west coast (Fig. 1). The system displays increasing eutrophication towards its inner parts, as documented, for example, by environmental monitoring data on nutrient levels, chlorophyll-*a*, and water transparency (Swedish Meteorological and Hydrological Institute, Supplementary material, S1). The area is also affected by long-term fishing pressure, causing significant depletion of many piscivorous fish, mainly gadoids (Svedäng and Bardon 2003; Cardinale and Svedäng 2004). In response to this, the whole area was closed to trawl fisheries in 2004, and after that also other gear type and catch restrictions have been imposed. Since 2010, all fishing targeting the main gadoid species (cod *Gadus morhua*; haddock *Melanogrammus aeglefinus*; pollack *Pollachius pollachius*; www.8fjordar.se) is prohibited in water bodies A, B, and C (Fig. 1). In addition to these anthropogenic impacts, there are differences in natural environmental conditions between water bodies in the fjord system, as well as small-scale gradients within these, for example in water depth, wave exposure, bottom temperatures, and vegetation cover. The ambient environmental gradients were quantified in connection with the field work and evaluated as described below.

### Sampling methods

The fish survey was conducted at a total of 300 stations in five subareas, covering a total area of 190 km^2^, from 8 August 2012 to 4 September 2012 (Fig. 1; Table 1). The stations were positioned by stratification within 0–10 m depth, so that 40 stations were randomised within the depth interval 0–6 m and 20 stations within 6–10 m in each subarea. Hence, data from 58 to 60 stations were obtained within each subarea (data from five stations were discarded due to disturbances during fishing). Fishing was carried out using fyke nets according to national monitoring standards (SwAM 2015). This method targets benthic and demersal fish species, which are the predominating groups of coastal fish fauna in the area. The fyke nets were 55 cm high with a semicircular opening, a 5-m-long arm, and a mesh size of 11 mm. Two connected fyke nets were set at each station. These were connected arm to arm, except for 20 stations in the shallower depth interval which were placed close to the shoreline and were connected arm to crib. The fyke nets were set in the afternoon and lifted in the morning in order to cover the period when the fish are most active. Catches were recorded directly on board as numbers per species and station.

**Table 1:** List of species encountered in the fish survey and their classification into mesopredator or other species in the present study

Name	Scientific name	TL	Range	Mean ± SE
Mesopredatory fish				
Shorthorn sculpin	*Myoxocephalus scorpius*	3.9	0–4	0.183 ± 0.033
Brill	*Scophthalmus rhombus*	3.8	0–2	0.007 ± 0.007
Longspined bullhead	*Taurulus bubalis*	3.6	0–6	0.111 ± 0.029
Fivebeard rockling	*Ciliata mustela*	3.5	0–1	0.031 ± 0.010
Three-spined stickleback	*Gasterosteus aculeatus*	3.5	0–1	0.003 ± 0.003
Eelpout	*Zoarces viviparus*	3.5	0–20	0.691 ± 0.102
Fifteen-spined stickleback	*Spinachia spinachia*	3.5	0–1	0.003 ± 0.003
Goldsinny wrasse	*Ctenolabrus rupestris*	3.4	0–93	2.844 ± 0.458
Greater pipefish	*Syngnathus acus*	3.4	0–1	0.007 ± 0.005
Rock cook	*Centrolabrus exoletus*	3.3	0–9	0.054 ± 0.032
Common dragonet	*Callionymus lyra*	3.3	0–1	0.003 ± 0.003
Plaice	*Pleuronectes platessa*	3.3	0–3	0.129 ± 0.024
Dab	*Limanda limanda*	3.3	0–1	0.003 ± 0.003
Corkwing wrasse	*Symphodus melops*	3.3	0–68	3.034 ± 0.546
Flounder	*Platichthys flesus*	3.2	0–7	0.329 ± 0.050
Black goby	*Gobius niger*	3.2	0–4	0.247 ± 0.037
Sole	*Solea solea*	3.1	0–4	0.061 ± 0.021
Ballan wrasse	*Labrus bergylta*	3.1	0–2	0.037 ± 0.014
Other fish species				
Saithe	*Pollachius virens*	4.4	0–17	0.566 ± 0.103
Cod	*Gadus morhua*	4.4	0–24	0.786 ± 0.129
Whiting	*Merlangius merlangus*	4.4	0–8	0.112 ± 0.035
Pollack	*Pollachius pollachius*	4.2	0–2	0.014 ± 0.008
European eel	*Anguilla anguilla*	3.5	0–36	1.725 ± 0.235
Sea trout	*Salmo trutta*	3.2	0–1	0.017 ± 0.007

Range = min − max value recorded at any station. Mean ± SE = total mean number of individuals per station (±standard error)

*TL* Trophic level (Froese and Pauly 2015)

Mesopredators were identified as species with a predominantly zoobenthic diet, as demonstrated based on stomach content analyses in most cases (Wennhage and Pihl 2002, Kraufvelin et al. in prep.). For the remaining species, the ones with a trophic level <4.0 according to Froese and Pauly (2015) were identified (Table 1). However, sea trout (*Salmo trutta*) and European eel (*Anguilla anguilla*) were not included as these are highly migratory and not typical coastal resident species.

### Environmental variables

Data on environmental variables were registered in connection with the fishing. The fished depth was noted, and water temperature (°C) and salinity (psu) were registered at the fished depth at each station. Water transparency was measured as the Secchi disc depth. In cases where the Secchi depth exceeded the actual water depth, stations were assigned a Secchi depth value by spatial interpolation based on the five most adjacent points, using inverse distance weighting in ArcGIS. In addition, wave exposure at each station was estimated using the software WaveImpact (Isæus 2004), which has been extensively used for predicting distribution patterns in coastal areas (e.g. Sundblad et al. 2011, 2014). Vegetation cover was surveyed using drop video, and the predominating vegetation was subsequently identified in the laboratory.

In order to test the research hypothesis, the information from drop video was transferred to a variable describing the cover of structurally complex vegetation (“Vegetation cover”; estimated to 0, 25, 50, 75, or 100 % cover; *cf* Heck and Orth 1980; Isaksson et al. 1994). Structurally complex vegetation was defined as eelgrass and upright coarse macroalgae (mainly genera *Fucus, Laminaria, Ascophyllum*). For loose-lying specimens and attached delicately branched filamentous species (Lobban and Harrison 1994; mainly *Ceramium* spp*., Polysiphonia* spp.), the per cent cover was set to 0. Water transparency was used as a proxy of eutrophication status (HELCOM 2007; Cloern 2001; Fleming-Lehtinen and Laamanen 2012). Even though other factors also affect water transparency, such as the amount of coloured dissolved organic matter and suspended particulate matter, there is a pronounced influence on the Secchi depth from chlorophyll-*a* in the Skagerrak area (Aas et al. 2014; Harvey 2015). Data on piscivorous fish were obtained from the fish survey. The biotic variable “Piscivores” was computed as the total number per station of sampled species that have been shown to be predominantly feeding on fish (Wennhage and Pihl 2002) and with a trophic level ≥4.0 (Froese and Pauly 2015) hence including cod, saithe (*Pollachius virens*), whiting (*Merlangius merlangus*), and pollack. In addition, the abundance of European eel was added as a potential explanatory variable (“Eel”), in order to explore its relationship to the mesopredatory fish species, given its relatively high abundance in the studied area. Temperature, salinity, depth, and wave exposure were considered as natural abiotic variables with a potential effect on the local distribution patterns of mesopredatory fish (Table 2).

**Table 2:** Environmental variables assessed and their categorisation in the analyses

Variable	Unit	Categorisation	Range	Mean ± SE
Salinity	(psu)	Natural abiotic	10.4–21.5	18.4 ± 0.1
Temperature	°C	Natural abiotic	15.6–20.6	18.2 ± 0.1
Depth	m	Natural abiotic	0.6–10.0	4.5 ± 0.2
Wave exposure	–	Natural abiotic	3.2–5.8	4.1 ± 0.1
Vegetation cover	%	Habitat quality	0–100	33.1 ± 2.3
Water transparency	m	Habitat quality	0.8–10.0	5.8 ± 0.1
Piscivores	n	Predation	0–5.4	0.7 ± 0.1
Eel	n	Predation	0–6.0	1.1 ± 0.1

Values give the range (min and max per station) and the total mean (±SE) in the study area

### Mesopredatory fish assemblages in relation to environmental variables

The association between the observed species composition and environmental variables was assessed using distance-based linear modelling, DISTLM (Legendre and Anderson 1999; McArdle and Anderson 2001). All fish species a priori identified as coastal mesopredators (Table 1) were included as response variables. Data were entered as numbers of individuals per station after square root transformation in order to balance the influence between more dominant and rare species. A dummy variable of 1 was added to the data set in order to cope with totally empty samples. The environmental variables were normalised prior to analyses. Wave exposure was also log_10_(*x* + 1)-transformed and the variables “Piscivores” and “Eel” were square root transformed in order to improve linearity. The level of correlation between each of the environmental variables was generally low (Pearson’s correlation coefficient < 0.53 for all pairs), and their variation inflation factors (VIFs; Zuur et al. 2010) were below 3. Differences in environmental variables between subareas were tested for by use of one-way ANOVAs with a posteriori SNK tests.

The DISTLM analyses are based on a resemblance matrix which shows the level of similarity in species composition among sampled stations. The resemblance matrix was based on the Bray–Curtis similarity index, which is well suited for species abundance data and avoids common absences to be treated as similarities. The analyses were performed in PERMANOVA+ as implemented in PRIMER version 6 (Anderson 2005; Anderson et al. 2008), using the “Best” option, which examines all possible combinations of predictor variables (Clarke and Gorley 2006). First, best models were identified for a series of alternative models including an increasing number of explanatory variables, from a model including only one variable to a model including all potential predictor variables. Then, the most parsimonious model among these was identified by the corrected Akaike information criterion AIC_c_. Alternative models differing from each other with less than 2 units of the selection criterion were identified as potential parallel models, and in these cases the variables with the lower number of variables included were preferred (Burnham and Anderson 2002). The final model was visualised by distance-based redundancy analysis, dbRDA (Legendre and Anderson 1999; McArdle and Anderson 2001), which is constrained to find linear combinations of predictor variables that explain the greatest variation in the data cloud (Anderson et al. 2008). The relative influence of each predictor variable was assessed based on the length of their overlaid vectors in the resulting ordination plot. In addition, the individual relationship of each environmental variable to the observed pattern in the species data set was identified based on marginal *F* tests.

### Mesopredatory fish abundance in relation to environmental variables

In addition, the univariate relationship between the abundance of mesopredatory fish and environmental variables was assessed. Based on the outcome of the multivariate analyses, the sum of all fish species defined as coastal mesopredators (Table 1) was used as the response variable. The potential explanatory variables were also the same as in the multivariate analyses, however, excluding “Eel” which was not identified as a key variable in the multivariate analyses. In addition, in order to account for the fact that most of the potential piscivores in the system had small body size and may not yet have reached the stage of having a piscivorous diet, the analyses were also performed separately with potential piscivores >30 cm length as an explanatory variable. The analyses were performed using generalised additive models (GAM) with a log link (quasi-Poisson) in order to suit the properties of the assessed data sets, as verified by initial exploration of the data. A smoothing function (*k* = 3) was used for all explanatory variables. Final models for selected variable combinations were identified using backward selection until only significant variables (*p* < 0.05) remained. In order to address the relative influence of habitat quality, nutrient status, and predation on species distribution and compare these with an alternative model based on the best combination of abiotic variables, the following variable combinations were addressed: (1) vegetation cover only, (2) water transparency only, (3) piscivores only, (4) abiotic factors only (including temperature, salinity, depth, and wave exposure). Subsequently, the relative improvement in the abiotic model by any of the other variables was addressed by adding them each at a time to the abiotic model. Finally, optimal models potentially including any variable combination were identified. The analyses were carried out in R 3.0.1 as implemented in Brodgar 2.7.4 (Highland Statistics).

## Results

### Biotic and environmental patterns

**Fig. 2:**
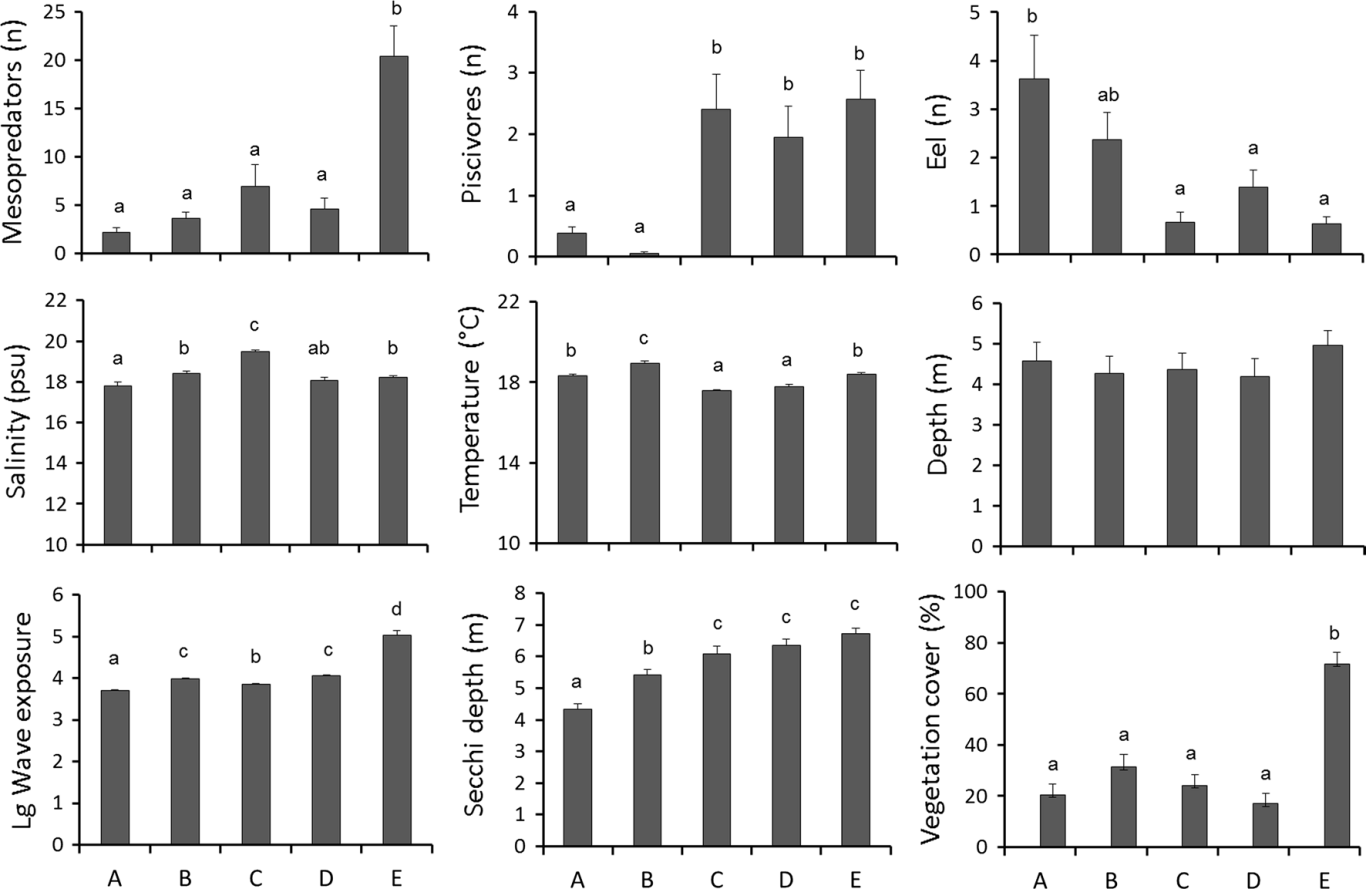
Distribution of the studied variables over the subareas *A*–*E*: *Upper row* abundance of mesopredatory fish, piscivores and eel; *Middle row* salinity, temperature, and depth; *Lower row* wave exposure, water transparency (Secchi depth), and vegetation cover. Values show mean values per station + standard error. Symbols identify significant differences among subareas

In total, 24 fish species were registered in the fish survey. Of these, 17 (71 %) were classified as mesopredators. These species constituted 69 % in terms of fish abundances (Table 1). Of the mesopredatory fish, 40 % were corkwing wrasse, 37 % goldsinny wrasse, 9 % eelpout (*Zoarces viviparus*), 4 % flounder (*Platichthys flesus*), and 3 % black goby (*Gobius niger*). The mesopredatory fish were most common in the outermost subarea (Fig. 2). Other frequent species in the catches were European eel (yellow eel stage, 16 % of total abundance) and gadoids (saithe, cod, whiting, and pollack, 13 % of total abundance). These showed inverse abundance patterns, with eel being relatively more abundant in the inner parts of the gradient, where gadoids were particularly scarce (Fig. 2). In subarea B, only three gadoids were caught (saithe). Most gadoids were small sized. Ten per cent of the gadoids were above 30 cm length, and all of these were cod.

According to one-way ANOVAs with a posteriori SNK test, there were no systematic differences in *temperature* or *salinity* from the inner to the outer subareas (Fig. 2). The most prominent changes along this gradient were seen for *vegetation cover*, *wave exposure,* and *water transparency*. High values in these variables were most frequent in the outermost subarea (E), although stations with low values also occurred (Table 2). Water temperature was highest in subarea B and lowest in C–D, whereas salinity was highest in subarea C and lowest in A.

### Multivariate biotic pattern in relation to environmental variables

**Fig. 3:**
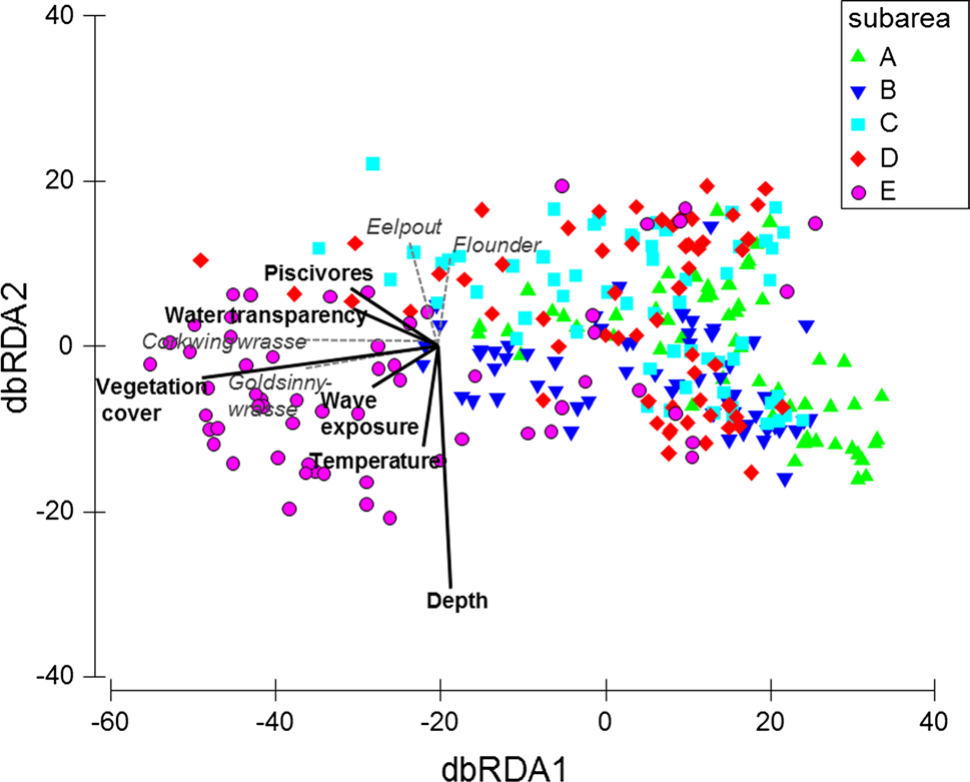
Multivariate relationship between the abundance patterns of mesopredator fish species and environmental variables, visualised by dbRDA. Environmental variables to include were selected by DISTLM (Table 3). Vectors show the relative contribution of each environmental variable and the direction and species exhibiting the strongest relationship to the environmental variables (longer vector indicate a stronger influence on the ordination). dbRDA1 explained 78.2 % of the fitted and 25.6 of the total variation in the data set. The second axis (dbRDA2) explained 15.3 % of the fitted and 5 % of the total variation

The DISTLM identified variables and combinations of environmental variables most likely to explain the observed distribution pattern of mesopredatory fish (Table 3). The most parsimonious model was identified for a combination of six environmental variables added in the following sequence: *vegetation cover*, *piscivores*, *depth*, *wave exposure*, *water transparency*, and *temperature*. Together, these variables accounted for 33.6 % of the total variation in the mesopredator data cloud. Marginal tests identified *vegetation cover* as the single variable that was most strongly attributed to changes in the mesopredator data cloud (explaining 19.5 % of total variation), followed by *wave exposure* (13.8 % explained; Table 3). The variables *salinity* and *eel* were not included. The outcome was visualised in a dbRDA (Fig. 3), which captured the main part of the variation explained by the model on the first axis (78.2 % of the fitted and 25.6 % of the total variation). The species showing the strongest influence on the observed patterns were corkwing wrasse and goldsinny wrasse. These species showed a positive relationship to *vegetation cover*, *water transparency,* and *wave exposure*, but also to *piscivores* (Fig. 3). The second axis encompassed a considerably smaller share of the explained variation (15.3 % of the fitted and 5 % of the total variation). This axis was mainly influenced by eelpout and flounder, which were both rarer in the data set than the corkwing and goldsinny wrasses (Table 1). These species primarily showed a negative relationship to *water temperature* and *depth.* Despite this deviation, the overall strong contribution of the first dbRDA axis was interpreted as an indication that the studied group of mesopredatory fish overall showed similar response to the studied environmental factors, and subsequent univariate analyses were conducted based on their total summed abundance (species identified in Table 1).

**Table 3:** Relationship between the mesopredatory fish species matrix and environmental variables according to the DISTLM

Variable	Categorisation	Included in (model nr)	Marginal test		% explained
			Pseudo-*F*	*p*	
Vegetation cover	Habitat quality	1,2,3,4,5,6	71.0	<0.001	19.5
Wave exposure	Natural abiotic	4,5,6	47.1	<0.001	13.8
Water transparency	Eutrophication	5,6	28.8	<0.001	8.9
Piscivores	Predation	2,3,4,5,6	20.1	<0.001	6.4
Depth	Natural abiotic	3,4,5,6	14.7	<0.001	4.8
Temperature	Natural abiotic	6	8.1	<0.001	2.7
Eel	Top-down		6.2	<0.001	2.1
Salinity	Natural abiotic		5.6	<0.001	1.9

Optimal variable combinations for models including an increasing number of variables were identified. Column 3 indicates in what final model each variable was included; 1 = model with one variable, 2 = model with two variables, etc. The overall best solution was identified as a model including six variables, based on the selection by AICc. Columns 4–6 give the result of marginal tests showing the individual importance of each environmental variable in determining the mesopredator species pattern

### Univariate relationships

**Fig. 4:**
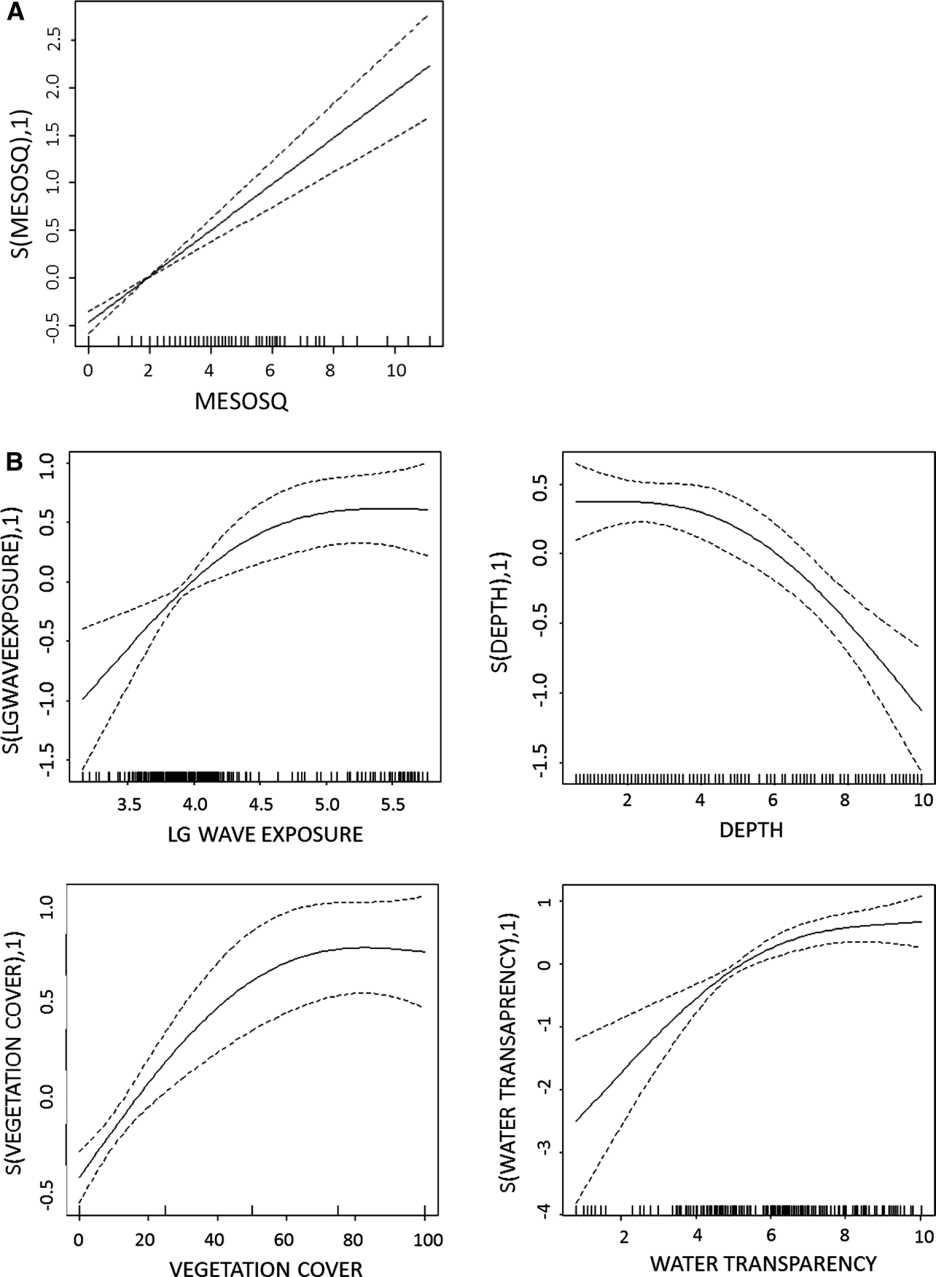
**a** Response curve for the assessment of piscivore abundance as a function of mesopredatory fish. **b** Response curves for the variables included in the optimised GAM model of mesopredator abundance; Wave exposure, depth, vegetation cover and water transparency. In all graphs, the *y* axis gives the relative effect of the smoother at different levels of the explanatory variable

The univariate analyses (GAM) of relationships to environmental variables identified a strong positive relationship between total mesopredator abundance and piscivores (Fig. 4a; Table 4), corresponding to the multivariate dbRDA output (Fig. 3). As this pattern did not reflect a predation effect on mesopredatory fish, piscivore abundance was not considered an explanatory variable in the further analyses. Instead, an alternative scenario was evident with piscivores being abundant in the same areas where mesopredator abundance is also highest, with a close to linear relationship (subsequent generalised linear model on the effect of mesopredatory fish on piscivore abundance: slope = 0.21, *t* = 7.95, *p* < 0.001, deviance explained = 17.7 %; Fig. 4a). A highly similar result was obtained when the analysis only included cod larger than 30 cm (GLM: slope = 0.34, *t* = 5.789, *p* < 0.001, deviance explained = 18.1 %),

**Table 4:** Generalised additive models of the relationship between total mesopredator abundance and environmental variables

Model	*R* ^2^ (adj)	DevE	Variables	edf	*F*	*p*
Piscivores only	–	–	s(PISC)	1.92	47.5	<0.001
Vegetation cover only	0.22	37.7	s(VEGCOVER)	1.85	73.6	<0.001
Water transparency only	0.09	19.8	s(WATERTR)	1.9	25.4	<0.001
Abiotic factors only, final selected model	0.26	38	s(DEPTH)	1.9	20.6	<0.001
			s(WAVEXP)	1.85	57.3	<0.001
Abiotic factors final model + vegetation cover	0.34	47.8	s(DEPTH)	1.84	11.8	<0.001
			s(WAVEXP)	1.83	15	<0.001
			s(VEGCOVER)	1.85	23.3	<0.001
Abiotic factors final model + water transparency	0.29	45.4	s(DEPTH)	1.89	28.5	<0.001
			s(WAVEXP)	1.84	34.2	<0.001
			s(WATERTR)	1.84	15.4	<0.001
Optimised model	0.36	52.8	s(DEPTH)	1.82	14.5	<0.001
			s(WAVEXP)	1.76	8.6	<0.001
			s(VEGCOVER)	1.86	19.3	<0.001
			s(WATERTR)	1.83	13.4	<0.001

*DevE* Deviance explained (%), *edf* Effective degrees of freedom

Of the remaining variables (after omitting piscivores), “vegetation cover” explained the highest proportion of the deviance, or 37.7 %, when the variables were applied individually. A model with only “water transparency” explained 19.8 % of the deviance. Both variables showed a positive relationship to mesopredator abundance. In the final GAM model when including only natural abiotic variables, depth and wave exposure were retained as explanatory variables, whereas the influences of salinity and temperature were not significant. The deviance explained by the model was similar to that of vegetation cover only (38 %). When “water transparency” and “vegetation cover” were added to the abiotic final model, each variable improved the model to a similar extent. Hence, the optimised model included all these four variables (Fig. 4b), explaining 52.8 % of the total deviance (Table 4).

## Discussion

The analyses showed that mesopredatory fish were mainly associated with sites with a high cover of structurally complex vegetation and a low degree of eutrophication, as measured by water transparency, and that they were not locally regulated by the presence of piscivores. The results indicate that habitat-related variables mainly regulate mesopredatory fish abundance in the area, with higher abundances of mesopredatory fish in areas associated with higher habitat quality. A positive relationship between mesopredatory fish and piscivore abundance further supported an overall importance of bottom-up processes.

The lack of a top-down relationship is likely explained by a depletion of local piscivore populations due to historically high fishing pressure (Svedäng and Bardon 2003; Cardinale and Svedäng 2004). Despite more recently imposed fishing restrictions, the regulatory effect of piscivores on mesopredatory fish is probably low. Around 10 % of the gadoids in the samples were represented by fish above 30 cm, which is an approximate size limit for cod to shift to a predominantly piscivorous diet (Daan 1973). The average ratio between piscivores and mesopredatory fish ranged from very low 0.01 (in subarea B) to 0.42 (in subarea D). Corresponding proportions for piscivores above 30 cm were from 0 (in subarea B) to 0.10 (in subarea A). It should be noted, however, that these estimates are specific to the applied sampling method and depth range (shallow areas above 10 m depth) and not to the subareas in total. Many larger sized gadoids are likely to reside in deeper and cooler areas of the studied fjord system as well and may also forage in shallow areas. However, since the fyke nets were in place over night and hence included important time periods for foraging during dusk and dawn, the relative distribution of the different fish groups across the areas is probably adequately represented.

Although the distribution of mesopredatory fish abundance was best explained by the cover of structurally complex vegetation, it was also related to water transparency, which was used as a proxy for eutrophication. Nutrient enrichment and the associated increase in production of ephemeral algae may stimulate the production of certain grazers such as amphipods (Kraufvelin et al. 2006a) and gastropods (Díaz et al. 2012) and in turn support mesopredator assemblages (Moksnes et al. 2008; Eriksson et al. 2009; Östman et al. 2016). However, in this study an inverse relationship was seen, and the results more likely reflected the eutrophication gradient indirectly via effects on habitat quality. Eutrophication is well known to stimulate ephemeral algal species and induce overgrowth of perennial species, which will also impair habitat conditions for fish (Pihl et al. 1996; Cloern 2001; Berger et al. 2004; Kraufvelin et al. 2006b). With regard to the natural abiotic factors, the study indicated the highest mesopredatory fish abundances at 0–6 m depth and increasing abundances up to a threshold of wave exposure, which further supports this view. Although highly energetic environments may impair the abilities of the fish to swim, there are considerable indirect effects of wave exposure on the local habitat types which may affect the level of food and shelter positively (Denny 1985; Leigh et al. 1987; Kraufvelin 2007; Norderhaug et al. 2012).

We also included European eel as a potential explanatory variable, but this did not contribute to explaining the variation in mesopredatory fish abundance. Thus, there was no negative relationship between the two species groups, although previous studies of eel have shown that the yellow eel stage of the European eel is a main feeder on mesopredatory fish (Costa et al. 1992; Moriarty 2003). Eel was mainly present in the inner estuarine parts of the fjord system and seemed to be related to other environmental factors than mesopredatory fish (Feunteun 2002). However, identifying factors affecting the local distribution of eel and its biotic relationships remains a topic for further studies.

The most common mesopredatory fish species were corkwing wrasse and goldsinny wrasse. The results corroborate those of other studies with respect to these species, showing a strong association to structurally rich environments where the wrasses find recruitment habitats, refuges from predation as well as prey (Sayer et al. 1995, 1996; Wennhage and Pihl 2002; Bergström et al. 2013; Skiftesvik et al. 2015). Our study provides a larger-scale perspective to these findings by comprising a large amount of data spanning over vast environmental gradients and by assessing the relative importance of different environmental factors. Typically, it is not possible to assess many environmental factors simultaneously in a detailed way in studies conducted at smaller geographical scale and over time. One deficiency in our study, however, was the lack of a strong gradient in piscivore abundances. The local abundance of piscivores was relatively low in all parts of the studied fjord system, a situation which is also true for surrounding coastal areas. Hence, the most promising way forward to assess this aspect further is probably to revisit the study area later in time, in order to follow up on the effects of a potential recovery of piscivores as a response to ongoing management regulations. Despite this shortcoming, the results serve to question the developing view that mesopredators increase in disrupted systems, as they in this study were more abundant in areas associated with good habitat quality and relatively higher piscivore abundances, as well.

### Management aspects

There is an increasing request to assess the environmental status of key ecosystem components and to assign indicators to monitor management performance. In Swedish coastal waters, mesopredatory fish are planned to be included in the evaluation of good environmental status of biodiversity and food webs within the European Marine Strategy Framework Directive (descriptors 1 and 4; EC 2008), to complement existing coastal status assessments which do not encompass coastal fish (Water Framework Directive (WFD; EC 2000; SwAM 2012). The relevance of mesopredatory fish for coastal management is additionally motivated by their increased interest for commercial fishery. Coastal mesopredatory fish are not traditional target species for fisheries in the region, but the demand is increasing from salmon aquaculture (ICES 2015). This study highlights the complex ways through which bottom-up and top-down effects may interact to configure food webs, showing that at low piscivore abundances, the distribution of coastal mesopredators and piscivores is tightly coupled. The importance of bottom-up factors for piscivore productivity emphasises the need to avoid overharvesting of fish at all trophic levels.

Our results support the inclusion of coastal mesopredatory fish as a quality element for assessing environmental status of coastal ecosystems. Mesopredatory fish abundance shows spatial variability at an assessment scale relevant for local management (water bodies) and is responsive to pressures rooted in human activities. Under the predominating environmental conditions of this study, a mesopredator indicator may be expected to show a positive relationship to food web functionality. Further studies should be directed towards identifying thresholds for good environmental status and defining the external conditions under which these are applicable. Changes in mesopredator abundance may be combined with information on piscivore abundance to assess the overall functionality of the coastal food webs. However, under predominating bottom-up conditions, as in the present case, we propose that mesopredators may also be monitored in their own sense as a measure of performance of the coastal habitat. As the coastal mesopredatory fish species are stationary, they provide a more precise indicator of local environmental conditions than piscivorous species which are typically more mobile. Monitoring mesopredators may also be preferential for ethical reasons, as surveys directly targeting piscivores may not be desirable or easily accomplished in areas where these are present in low numbers, threatened or protected.

## Conclusions

The results of the multivariate and the univariate analyses were highly consistent, indicating that higher mesopredatory fish abundances mainly occurred in areas associated with high habitat quality, represented by high cover of large habitat-forming vegetation and lower degree of eutrophication. Indications of predation control were not observed, which was probably explained by the low levels of piscivores. Top-down control of mesopredators may be expected in areas with higher abundances of piscivores, but such areas are currently not available in the region due to overfishing. On the other hand, mesopredators were not associated with disturbed areas and increased eutrophication symptoms, such as impoverished perennial vegetation and decreased water transparency, as may be expected in areas affected by cascading effects from a loss of piscivorous fish. Sites with higher abundance of mesopredators also had relatively higher abundances of piscivores, mainly juveniles. Hence, the study underlines the importance of ensuring good habitat quality to allow for sustainable populations of mesopredatory fish as well as to support the recovery of piscivores and their associated ecosystem services.

## Electronic supplementary material

Below is the link to the electronic supplementary material.

Supplementary material 1 (PDF 40 kb)

## References

[CR1] Aas E, Høkedal J, Sørensen K (2014). Secchi depth in the Oslofjord-Skagerrak area: theory, experiments and relationships to other quantities. Ocean Sci.

[CR2] Airoldi L, Beck MW (2007). Loss, status and trends for coastal marine habitats of Europe. Oceanogr Mar Biol Annu Rev.

[CR3] Anderson MJ (2005). Permutational multivariate analysis of variance.

[CR4] Anderson MJ, Gorley RN, Clarke RK (2008). PERMANOVA + for PRIMER: guide to software and statistical methods.

[CR5] Baden S, Gullström M, Lunden B, Pihl L, Rosenberg R (2003). Vanishing seagrass (*Zostera marina* L.) in Swedish coastal waters. Ambio.

[CR6] Baden S, Emanuelsson A, Pihl L, Svensson CJ, Åberg P (2012). Shift in seagrass food web structure over decades is linked to overfishing. Mar Ecol Prog Ser.

[CR7] Berger R, Bergström L, Kautsky L (2004) How does eutrophication affect different life stages of *Fucus vesiculosus* in the Baltic Sea? In Kautsky H, Snoeijs P (eds) Biology of the Baltic Sea. Hydrobiologia 514:243–248

[CR8] Bergström L, Sundqvist F, Bergström U (2013). Effects of an offshore wind farm on temporal and spatial patterns in the demersal fish community. Mar Ecol Prog Ser.

[CR9] Britten GL, Dowd M, Minto C, Ferretti F, Boero F, Lotze HK (2014). Predator decline leads to decreased stability in a coastal fish community. Ecol Lett.

[CR10] Burnham KP, Anderson DR (2002). Model selection and multi-model inference: a practical information-theoretic approach.

[CR11] Byström P, Bergström U, Hjälten A, Ståhl S, Jonsson D, Olsson J (2015). Declining coastal piscivore populations in the Baltic Sea: where and when do sticklebacks matter?. Ambio.

[CR12] Cardinale M, Svedäng H (2004). Modelling recruitment and abundance of Atlantic cod, *Gadus morhua*, in the eastern Skagerrak-Kattegat (North Sea): evidence of severe depletion due to a prolonged period of high fishing pressure. Fish Res.

[CR13] Casini M, Cardinale M, Hjelm J, Vitale F (2005). Trends in CPUE and related changes in spatial distribution of demersal fish species in the Kattegat and Skagerrak, eastern North Sea, between 1981 and 2003. ICES J Mar Sci.

[CR14] Casini M, Lövgren J, Hjelm J, Cardinale M, Molinero JC, Kornilovs G (2008). Multi-level trophic cascades in a heavily exploited open marine ecosystem. Proc R Soc Lond B Biol.

[CR15] Clarke KR, Gorley RN (2006). PRIMER v.6: user manual/tutorial.

[CR16] Cloern JE (2001). Our evolving conceptual model of the coastal eutrophication problem. Mar Ecol Prog Ser.

[CR17] Costa JL, Assis CA, Almeida PR, Moreira FM, Costa MJ (1992). On the food of the European eel, *Anguilla anguilla* (L.), in the upper zone of the Tagus estuary Portugal. J Fish Biol.

[CR18] Costanza R, de Groot R, Sutton P, van der Ploeg S, Anderson SJ, Kubiszewski I, Farber S, Turner RK (2014). Changes in the global value of ecosystem services. Glob Environ Change.

[CR19] Daan N (1973). A quantitative analysis of the food intake of North Sea cod *Gadus morhua*. Neth J Sea Res.

[CR20] Daan N, Gislason H, Pope JG, Rice JC (2005). Changes in the North Sea fish community: evidence of indirect effects of fishing?. ICES J Mar Sci.

[CR21] Darwall WRT, Costello MJ, Donnelly R, Lysaght S (1992). Implication of life-history strategies for a new wrasse fishery. J Fish Biol.

[CR22] de Groot R, Brander L, van der Ploeg S, Costanza R, Bernard F, Braat L, Christie M, Crossman N, Ghermandi A, Hein L, Hussain S, Kumar P, McVittie A, Portela R, Rodriguez LC, ten Brink P, van Beukering P (2012). Global estimates of the value of ecosystems and their services in monetary units. Ecosyst Serv.

[CR23] Denny MW (1985). Wave-forces on intertidal organisms—a case study. Limnol Oceanogr.

[CR24] Díaz ER, Kraufvelin P, Erlandsson J (2012). Combining gut fluorescence technique and spatial analysis to determine *Littorina littorea* grazing dynamics in nutrient-enriched and nutrient-unenriched littoral mesocosms. Mar Biol.

[CR25] EC (2000) Directive 2000/60/EC of the European Parliament and of the Council of 23 October 2000 establishing a framework for Community action in the field of water policy. Official Journal of the European Communities, L327

[CR26] EC (2008) Directive 2008/56/EC of the European Parliament and of the Council of 17 June 2008 establishing a framework for community action in the field of marine environmental policy (Marine Strategy Framework Directive). Official Journal of the European Union, L164

[CR27] Eriksson BK, Ljunggren L, Sandström A, Johansson G, Mattila J, Rubach A, Råberg S, Snickars M (2009). Declines in predatory fish promote bloom-forming macroalgae. Ecol Appl.

[CR28] Eriksson BK, Sieben K, Eklöf J, Ljunggren L, Olsson J, Casini M, Bergström U (2011). Effects of altered offshore food webs on coastal ecosystems emphasize the need for cross-ecosystem management. Ambio.

[CR29] Estes JA, Terborgh J, Brasheres JS, Power MS, Berger J, Bond WJ, Carpenter SR, Essington TE, Holt RD, Jackson JBC, Marquis RJ, Oksanen L, Oksanen T, Paine RT, Pikitch ET, Ripple WJ, Sandin SA, Scheffer M, Schoener T, Shurin JB, Sinclair ARE, Soulé ME, Virtanen R, Wardle DA (2011). Trophic downgrading of planet earth. Science.

[CR30] Feunteun E (2002). Management and restoration of European eel population (*Anguilla anguilla*): an impossible bargain. Ecol Eng.

[CR31] Fleming-Lehtinen V, Laamanen M (2012). Long-term changes in Secchi depth and the role of phytoplankton in explaining light attenuation in the Baltic Sea. Estuar Coast Shelf Sci.

[CR32] Frank KT, Petrie B, Choi JS, Leggett WC (2005). Trophic cascades in a formerly cod-dominated ecosystem. Science.

[CR33] Froese R, Pauly D (eds) (2015) FishBase. World Wide Web electronic publication. www.fishbase.org

[CR34] Gjøsæter J, Paulsen Ø (2004) Strandnotundersøkelser på Skagerrakkysten 2003. Havforskningsinstituttet, Report (**In Norwegian**)

[CR35] Halpern BS, Walbridge S, Selkoe KA, Kappel CV, Micheli F, D’Agrosa C, Bruno JF, Casey KS, Ebert C, Fox HE, Fujita R, Heinemann D, Lenihan HS, Madin EMP, Perry MT, Selig ER, Spalding M, Steneck R, Watson R (2008). A global map of human impact on marine ecosystems. Science.

[CR36] Harvey T (2015) Bio-optics, satellite remote sensing and Baltic Sea ecosystems: applications for monitoring and management. Ph.D. thesis, Stockholm University, p 58

[CR37] Heck KL, Orth RJ, Kennedy VS (1980). Seagrass habitats: the roles of habitat complexity, competition and predation in structuring associated fish and motile macroinvertebrate assemblages. Estuarine perspectives.

[CR38] HELCOM (2007) HELCOM Baltic Sea action plan. Adopted by the HELCOM ministerial meeting. Krakow, Poland 15th November 2007

[CR39] International Council for Exploration of the Sea (ICES) (2015) Catch statistics. Access date May 2015. http://www.ices.dk/marine-data/dataset-collections/Pages/Fish-catch-and-stock-assessment.aspx

[CR40] Isæus M (2004) Factors structuring *Fucus* communities at open and complex coastlines in the Baltic Sea. Ph.D. thesis, Stockholm University

[CR41] Isaksson I, Pihl L, van Montfrans J (1994). Eutrophication-related changes in macrovegetation and foraging of young cod (*Gadus morhua* L.): a mesocosm experiment. J Exp Mar Biol Ecol.

[CR42] Jackson JB, Kirby MX, Berger WH, Bjorndal KA, Botsford LW, Bourque BJ, Bradbury RH, Cooke R, Erlandson J, Estes JA, Hughes TP, Kidwell S, Lange CB, Lenihan HS, Pandolfi JM, Peterson CH, Steneck RS, Tegner MJ, Warner RR (2001). Historical overfishing and the recent collapse of coastal ecosystems. Science.

[CR43] Kraufvelin P (2007). Responses to nutrient enrichment, wave action and disturbance in rocky shore communities. Aquat Bot.

[CR44] Kraufvelin P, Salovius S, Christie H, Moy FE, Karez R, Pedersen MF (2006). Eutrophication-induced changes in benthic algae affect the behaviour and fitness of the marine amphipod *Gammarus locusta*. Aquat Bot.

[CR45] Kraufvelin P, Moy FE, Christie H, Bokn TL (2006). Nutrient addition to experimental rocky shore communities revisited: delayed responses, rapid recovery. Ecosystems.

[CR46] Legendre P, Anderson MJ (1999). Distance-based redundancy analysis: testing multispecies responses in multifactorial ecological experiments. Ecol Monogr.

[CR47] Leigh EG, Paine RT, Quinn JF, Suchanek TH (1987). Wave energy and intertidal productivity. Proc Natl Acad Sci USA.

[CR48] Lobban CS, Harrison PJ (1994). Seaweed ecology and physiology.

[CR49] McArdle BH, Anderson MJ (2001). Fitting multivariate models to community data: a comment on distance-based redundancy analysis. Ecology.

[CR50] Moksnes PO, Gullström M, Tryman K, Baden S (2008). Trophic cascades in a temperate seagrass community. Oikos.

[CR51] Möllmann C, Diekmann R, Müller-Karulis B, Kornilovs G, Plikshs M, Axe P (2009). Reorganization of a large marine ecosystem due to atmospheric and anthropogenic pressure: a discontinuous regime shift in the Central Baltic Sea. Glob Change Biol.

[CR52] Moriarty C, Aida K, Tsukamoto K, Yamauchi K (2003). The yellow eel. Eel biology.

[CR53] Moy FE, Christie H (2012). Large-scale shift from sugar kelp (*Saccharina latissima*) to ephemeral algae along the south and west coast of Norway. Mar Biol Res.

[CR54] Norderhaug KM, Christie H, Andersen GS, Bekkby T (2012). Does the diversity of kelp forest macrofauna increase with wave exposure?. J Sea Res.

[CR55] Östman Ö, Eklöf J, Eriksson BK, Olsson J, Moksnes P-O, Bergström U (2016). Top-down control as important as nutrient enrichment for eutrophication effects in North Atlantic coastal ecosystems. J Appl Ecol.

[CR56] Pauly D, Christensen V, Dalsgaard J, Froese R, Torres F (1998). Fishing down marine food webs. Science.

[CR57] Pihl L, Wennhage H (2002). Structure and diversity of fish assemblages on rocky and soft bottom shores on the Swedish west coast. J Fish Biol.

[CR58] Pihl L, Magnusson G, Isaksson I, Wallentinus I (1996). Distribution and growth dynamic of ephemeral macroalgae in shallow bays on the Swedish west coast. J Sea Res.

[CR59] Rönnbäck P, Kautsky N, Pihl L, Troell M, Söderqvist T, Wennhage H (2007). Ecosystem goods and services from Swedish coastal habitats: identification, valuation, and implications of ecosystem shifts. Ambio.

[CR60] Sayer MDJ, Gibson RN, Atkinson RJA (1995). Growth, diet and condition of goldsinny on the west coast of Scotland. J Fish Biol.

[CR61] Sayer MDJ, Gibson RN, Atkinson RJA (1996). Growth, diet and condition of corkwing wrasse and rock cook on the west coast of Scotland. J Fish Biol.

[CR62] Seitz RD, Wennhage H, Bergström U, Lipcius RN, Ysebaert T (2014). Ecological value of coastal habitats for commercially and ecologically important species. ICES J Mar Sci.

[CR63] Sieben K, Ljunggren L, Bergström U, Eriksson BK (2011). A meso-predator release of stickleback promotes recruitment of macroalgae in the Baltic Sea. J Exp Mar Biol Ecol.

[CR64] Skiftesvik AB, Blom G, Agnalt AL, Durif CMF, Browman HI, Bjelland RM, Harkestad LS, Farestveit E, Paulsen OI, Fauske M, Havelin T, Johnsen K, Mortensen S (2014). Wrasse (Labridae) as cleaner fish in salmonid aquaculture—the Hardangerfjord as a case study. Mar Biol Res.

[CR65] Skiftesvik AB, Durif CM, Bjelland RM, Browman HI (2015). Distribution and habitat preferences of five species of wrasse (Family Labridae) in a Norwegian fjord. ICES J Mar Sci.

[CR66] Stål J, Paulsen S, Pihl L, Rönnbäck P, Söderqvist T, Wennhage H (2008). Coastal habitat support to fish and fisheries in Sweden: integrating ecosystem functions into fisheries management. Ocean Coast Manage.

[CR67] Sundblad G, Bergström U, Sandström A (2011). Ecological coherence of marine protected area networks: a spatial assessment using species distribution models. J Appl Ecol.

[CR68] Sundblad G, Bekkby T, Isæus M, Nikolopoulos A, Norderhaug KM, Rinde E (2014). Comparing the ecological relevance of four wave exposure models. Estuar Coast Shelf Sci.

[CR69] Svedäng H, Bardon G (2003). Spatial and temporal aspects of the decline in cod (*Gadus morhua* L.) abundance in the Kattegat and eastern Skagerrak. ICES J Mar Sci.

[CR70] Swedish Agency for Water and Marine Management (SwAM) (2015) Undersökningstyp: Djupstratifierat provfiske med småryssjor (**in Swedish**)

[CR71] Swedish Agency of Water Management (SwAM) (2012) God Havsmiljö 2020, Del 2”God miljöstatus och miljökvalitetsnormer” (**in Swedish**). https://www.havochvatten.se/download/18.2a9b232013c3e8ee03e3c17/1362737191111/rapport-2012-20-god-havsmiljo-del-2.pdf

[CR72] Wennhage H, Pihl L (2002). Fish feeding guilds in shallow rocky and soft bottom areas on the Swedish west coast. J Fish Biol.

[CR73] Zuur AF, Ieno EN, Elphick CS (2010). A protocol for data exploration to avoid common statistical problems. Meth Ecol Evol.

