# Supplementary material for: Distribution of mesopredatory fish determined by habitat variables in a predator-depleted coastal system
Source: Mar Biol. 2016 Sep 7;163(10):201. doi: 10.1007/s00227-016-2977-9 (PMC5014906; doi:10.1007/s00227-016-2977-9)
Supplement: Supplementary file 1 — Supplementary material 1 (PDF 40 kb) [file 227_2016_2977_MOESM1_ESM.pdf]

Distribution of mesopredatory fish determined by habitat variables in a predator-depleted coastal system

Lena Bergström[1*], Martin Karlsson[1], Ulf Bergström[1], Leif Pihl[2], Patrik Kraufvelin[1,3]

1. Department of Aquatic Resources, Institute of Coastal Research, Swedish University of Agricultural Sciences, Skolgatan 6, SE-74242 Öregrund, Sweden

2. Department of Biological and Environmental Sciences, Gothenburg University, Kristineberg 566, 45178 Fiskebäckskil, Sweden.

3. Environmental and Marine Biology, Department of Biosciences, Åbo Akademi University, Artillerigatan 6, FI-20520 Åbo/Turku, Finland

*= Corresponding author: lena.bergstrom@slu.se

Fig. S1. Hydro-chemical characterization of the studied system during the year of study, based on official environmental monitoring data. Mean surface values at 0-10 m. Data based on sampling at three stations within each sub-area, evenly distributed over May-Aug. Sub-area A was sampled at 3 occasions, sub-areas B-E at 6 occasions. Error bars give standard deviation. (SMHI 2015).

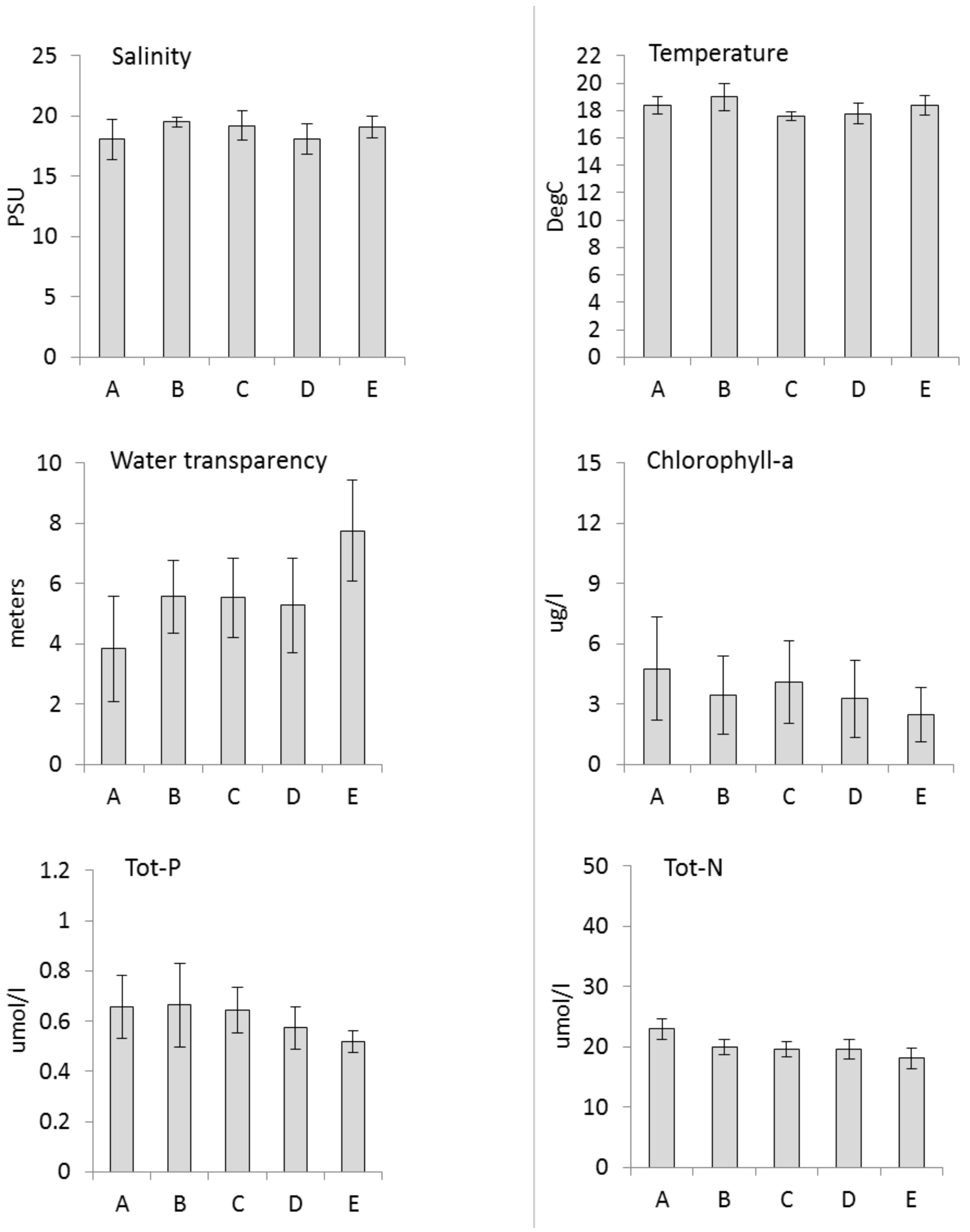
Salinity
PSU
0
5
10
15
20
25
A
B
C
D
E
Temperature
DegC
0
2
4
6
8
10
12
14
16
18
20
22
A
B
C
D
E
Water transparency
meters
0
2
4
6
8
10
A
B
C
D
E
Chlorophyll-a
ug/l
0
3
6
9
12
15
A
B
C
D
E
Tot-P
umol/l
0
0.2
0.4
0.6
0.8
1
1.2
A
B
C
D
E
Tot-N
umol/l
0
10
20
30
40
50
A
B
C
D
E
